# Characterization of Four Complete Mitogenomes of *Monolepta* Species and Their Related Phylogenetic Implications

**DOI:** 10.3390/insects15010050

**Published:** 2024-01-11

**Authors:** Rong-Rong Gao, Qi-Long Lei, Xu Jin, Iqbal Zafar, Xing-Ke Yang, Cheng-Yong Su, Jia-Sheng Hao, Rui-E Nie

**Affiliations:** 1 Anhui Provincial Key Laboratory of the Conservation and Exploitation of Biological Resources, College of Life Sciences, Anhui Normal University, Wuhu 241000, China; gaorongrong@ahnu.edu.cn (R.-R.G.); jinxu@ahnu.edu.cn (X.J.); iqzafarshah@gmail.com (I.Z.); sky475342@163.com (C.-Y.S.); 2 Department of Entomology, China Agricultural University, Beijing 100193, China; leiql@cau.edu.cn; 3 Guangdong Key Laboratory of Animal Conservation and Resource Utilization, Guangdong Public Laboratory of Wild Animal Conservation and Utilization, Guangdong Institute of Applied Biological Resources, Guangzhou 510260, China

**Keywords:** Coleoptera, *Monolepta*, high-throughput sequencing, mitochondrial genome, phylogeny

## Abstract

**Simple Summary:**

*Monolepta* is one of the largest groups in the subfamily Galerucinae, which has considerable ecological and economic significance. However, the lack of mitogenomic data for *Monolepta* limits our understanding of the taxonomy and phylogeny of this genus. Here, the completed mitogenomes of four *Monolepta* species were obtained using high-throughput sequencing technology. We compared the main features of newly sequenced mitogenomes, as well as the rate of evolution, base compositions, and relative synonymous codon usage (RSCU) of the mitogenomes among *Monolepta* species. Furthermore, combined with all available mitochondrial genomes and *ND1* data, the relationships of the section “Monoletites” at the suprageneric and species levels were explored. The section “Monoleptites” was proved to be a monophyletic group, while *Monolepta* was a non-monophyletic group. This study supported that the characteristic of “antennal segment 2 equals 3” of the true “*Monolepta*” evolved multiple times in several subgroups. This study will provide the basal data for further study of the taxonomy and phylogeny of Galerucinae.

**Abstract:**

*Monolepta* is one of the diverse genera in the subfamily Galerucinae, including 708 species and 6 sub-species worldwide. To explore the information on the mitogenome characteristics and phylogeny of the section “Monoleptites”, especially the genus *Monolepta*, we obtained the newly completed mitochondrial genomes (mitogenomes) of four *Monolepta* species using high-throughput sequencing technology. The lengths of these four new mitochondrial genomes are 16,672 bp, 16,965 bp, 16,012 bp, and 15,866 bp in size, respectively. All four mitochondrial genomes include 22 transfer RNA genes (*tRNAs*), 13 protein-coding genes (*PCGs*), 2 ribosomal RNA genes (*rRNAs*), and one control region, which is consistent with other Coleoptera. The results of the nonsynonymous with synonymous substitution rates showed that *ND6* had the highest evolution rate, while *COI* displayed the lowest evolution rate. The substitution saturation of three datasets (13 *PCGs*_codon1, 13 *PCGs*_codon2, 13 *PCGs*_codon3) showed that there was no saturation across all datasets. Phylogenetic analyses based on three datasets (*ND1*, 15 genes of mitogenomes, and *13 PCGs*_AA) were carried out using maximum likelihood (ML) and Bayesian inference (BI) methods. The results showed that mitogenomes had a greater capacity to resolve the main clades than the *ND1* gene at the suprageneric and species levels. The section “Monoleptites” was proven to be a monophyletic group, while *Monolepta* was a non-monophyletic group. Based on *ND1* data, the newly sequenced species whose antennal segment 2 was shorter than 3 were split into several clades, while, based on the mitogenomic dataset, the four newly sequenced species had close relationships with *Paleosepharia*. The species whose antennal segment 2 was as long as 3 were split into two clades, which indicated that the characteristic of “antennal segment 2 as long as 3” of the true “*Monolepta*” evolved multiple times in several subgroups. Therefore, to explore the relationships among the true *Monolepta*, the most important thing is to perform a thorough revision of *Monolepta* and related genera in the future.

## 1. Introduction

The genus *Monolepta* (Chevrolat, 1837) [1] is one of the largest genera of leaf beetles that belongs to the section “Monoleptites” in the subfamily Galerucinae (Coleoptera: Chrysomelidae), including 708 species and 6 sub-species worldwide [2]. In the Oriental region, 342 species were distributed, occupying almost half the species in this genus. In China, 73 species have been described, only 2 species distributing in the Palaearctic region, and 71 species in the Oriental region [3]. Both their larvae and adults are phytophagous, with most larvae living in soil and feeding on plant roots, and adults feeding on plant stems and leaves. Several well-known agricultural pest species belonging to this genus (e.g., *M. hieroglyphica* and *M. signata*) cause serious losses of some crops, such as soybean, corn, and rice, as well as some vegetables, around the world [4]. 

The section “Monoleptites”, established by Chapuis (1875), includes 36 genera in the tribe Luperini [5,6]. This section is separated from other sections of the tribe Luperini because it harbors a first hind tarsal segment that is distinctly longer than the remainder combined [7,8]. *Monolepta* is the biggest genus of the section “Monoleptites”, which is very complicated. There are two types (type I and type II) of antennae in *Monolepta*: in type I, segment 2 is equal to segment 3; in type II, segment 3 is longer than segment 2. Recently, much review work has been conducted by Wagner. Wagner re-described the type species *Monolepta bioculata* (Faricius, 1781) and re-checked many generic characteristics of *Monolepta*. *Monolepta* was considered to have closed anterior coxae cavities, but after the examination of the type species, it is known to have open rather than closed cavities [9]. In addition, there are no obvious concavities on the pronotum, and antennal segments 2 and 3 are equal in length. So, the “true” *Monolepta* belongs to antennae type II. Due to ‘unstable’ morphological characteristics and no description of male aedeagus, many species no longer belong to this genus. For example, eleven genera from Afrotropical and Oriental regions were established from the original. *Monolepta* is based on the characteristics of an anterior coxae, the ratio of its antennal length, and the convex on the pronotum and aedeagus [10,11,12,13,14,15,16,17,18]. As a result, 180 species are recorded from the African region. In China, research on *Monolepta* mainly focuses on its taxonomy and phylogeny. Yang et al. [3] provided a comprehensive catalog and species key of *Monolepta*. After 2015, several species were reviewed and new species were described: four species from Taiwan were transferred to genus *Paleosepharia*, Laboissière, 1936 [19], including *M. formosana* Chûjô, 1935 [20]; *M. amiana* Chûjô, 1962 [21]; *M. yasumatsui* Kimoto, 1969 [22]; and *M. nantouensis* Kimoto, 1996 [23]. *M. sublata* (Gressitt & Kimoto, 1963) [24] was designated as the type species of genus *Chinochya* Lee, 2020 [25]; *M. tsoui* Lee, 2009 [26] was transferred to the new genus *Tsouchya* Lee, 2020 [25]; five new species were described [27]; and *M. hieroglyphica* and *M. quadriguttata* became synonyms of *M. signata* [28]. 

The phylogenetic relationship of *Monolepta* at the species level has been explored by many researchers. Bolz and Wagner [29] first explored the phylogenetic relationship of “Monoleptites” including 16 species from *Monolepta*, using 20 external morphological characters and 14 male and female external genitalia features. The result showed that *Monolepta* was polyphyletic, with its four species standing out from most *Monolepta* species, that is, *M. versicolora* was closed to a clade of (*Galerudolphia* + *Barombiella*), and *M. duplicata*, *M. didyma*, and *M. thomsoni* were closed to *Candezea centromaculata*. Stapel et al. [8] explored the phylogenetic status of Afrotropical galerucines, including 14 species from *Monolepta*, based on morphological and molecular data (*ND1* and *ITS2*), and the results supported that *Monolepta* was polyphyletic and, additionally, indicated that an elongated metatarsus has evolved multiple times in Galerucinae. Nie et al. [30] used mitochondrial genomes, including from five species of “Monoleptites” which showed *Monolepta* were paraphyletic, too. 

The mitochondrial genome was a very powerful marker to explore the phylogeny of the Coleoptera in different ranks [31,32,33,34,35,36,37]. However, only six complete mitogenomes of *Monolepta* have been released by the NCBI database (https://www.ncbi.nlm.nih.gov/, accessed on 1 January 2024). The lack of available data severely limits comprehension of the classification and phylogenetic relationship of the genus *Monolepta*. In this study, four complete mitochondrial genomes were newly obtained. Firstly, we compared the main features, evolutionary rate, base compositions, and relative synonymous codon usage (RSCU) of whole mitochondrial genomes among *Monolepta* species. Then, we combined this with all available mitochondrial genomes and *ND1* data to reconstruct the phylogenetic relationship of the section “Monoletites” at the suprageneric and species levels. 

## 2. Materials and Methods

### 2.1. Taxon Sampling and DNA Extraction

The four adult samples were collected from different locations in China and preserved in absolute ethanol at −20 °C before DNA extraction. Genomic DNA was extracted from the head and prothorax of each specimen with a DNeasy Blood and Tissue kit (QIAGEN, Beijing, China) and eluted in 150 μL TE buffer, then kept at −80 °C until used. All newly sequenced species were identified by Professor Xing-Ke Yang and Dr. Qi-Long Lei. The voucher samples of the four taxa were kept at the Anhui Provincial Key Laboratory of the Conservation and Exploitation of Biological Resources, College of Life Sciences, Anhui Normal University.

### 2.2. Sequencing and Assembly

The genomic DNA was used to sequence the mitochondrial genomes using high-throughput sequencing on the Illumina Novo 6000 platform at Berry Genomics Corporation (Beijing, China) and libraries with 150 bp paired-end sequencing and 350 bp insert size sequencing. The principle of library preparation for sequencing was prepared using one sample with one library. The software Trimmomatic v.0.36 was used to trim the adapters [38]. Then, prinseq was used to remove low-quality and short reads [39]. Getorganelle v.1.7.7.0 was used for de novo assembly with high-quality reads under k-mer sizes of 21, 45, 65, 85, 105 and t-value 15 [40]. The gene annotations, checking for circularization, and extracting the individual protein-coding genes were performed in Geneious Prime 2020.2.4 [41]. We also used CGView Server (http://cgview.ca, accessed on 1 October 2023) to draw a map of the mitogenomes [42]. The formulas to calculate AT-skew and GC-skew were AT-skew = [A% − T%] / [A% + T%] and GC-skew = [G% − C%] / [G% + C%] [43]. The codon usage and relative synonymous codon usage of 13 *PCGs* were calculated by Phylosuite [44,45]. Calculating the rate of nonsynonymous (Ka) to synonymous (Ks) substitutions of 13 *PCGs* was performed by DnaSP 6.0 (Barcelona, Spain) [46]. Base compositions of the mitochondrial genome were analyzed in MEGA v.11 [47]. MITOS Web Server (http://mitos2.bioinf.uni-leipzig.de/index.py, accessed on 1 November 2023) was used to forecast the secondary structures and identify the anticodons of the *tRNAs* from the mitochondrial genomes [48]. The test of substitution saturation, with three datasets (13 *PCGs*_codon1, 13 *PCGs*_codon2, 13 *PCGs*_codon3), was performed by DAMGE v.7 with the GTR model [49].

### 2.3. Phylogenetic Analyses

The phylogenetic analyses of *Monolepta* species were carried out based on three types of datasets: (1) *ND1* gene from 34 taxa, using *Exosoma* sp. as an outgroup (accession number: AY116139) (Table S1); (2) 15 genes (13 *PCGs* and 2 *rRNAs*) from all available sixteen species of “Monoleptites” using two *Oides* species as outgroups (accession number: MF946622, MF960098) (Table 1); (3) 13 *PCG* amino acids (13 *PCGs*_AA) from 18 species, which is the same as in (2). Nucleotide sequences of 13 *PCGs* were aligned with TransAlign [50]. Two *rRNAs* and thirteen *PCGs*_AA were aligned with MUSCLE v.3.8.31 [51]. Using Gblocks 0.91, we selected conserved blocks from multiple alignments to filter the gaps and ambiguous sites in sequences under default parameters [52]. The aligned genes were concatenated by SequenceMatrix v.1.9 [53]. Under Bayesian inference (BI), we used Phylobayes MPI v1.5a under the CAT-GTR model for all searches to perform phylogenetic inferences [54]. Two parallel and independent tree searches were performed until the discrepancies were lower than 0.1 (maxdiff less than 0.1). A consensus tree was computed using the remaining trees from two runs after the initial 25% trees were discarded as burn-in. IQ-TREE v.2 was used to reconstruct the phylogenetic tree under maximum likelihood (ML) optimization [55]. The MFP-MERGE model was used for the bootstrapping phase and node support in all ML analyses was calculated by using 1000 SH-aLRT replicates [56] and 1000 UFBoot2 bootstraps (-B 1000, -alrt 1000), respectively [57]. 

## 3. Results

### 3.1. Sequence Data, Mitogenomic Organization, and Composition in Monolepta

**Figure 1:**
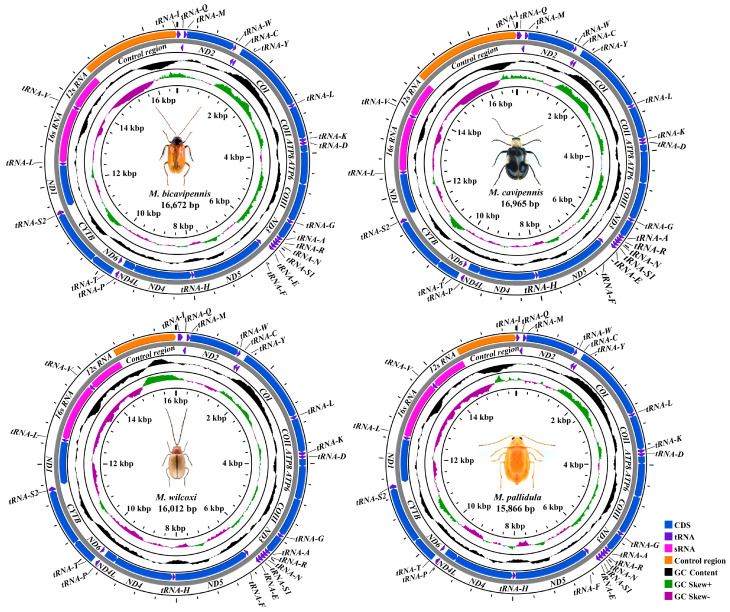
Circle maps of the four complete mitochondrial genomes of *Monolepta* species, with different colors to distinguish different genes.

Raw reads (about 15 Gb) were obtained for each sample using high-throughput sequencing technology. A total of four newly sequenced complete mitogenomes of *Monolepta* were obtained in this study, which were *M. bicavipennis* Chen, 1942 [60]; *M. cavipennis* (Baly, 1878) [61]; *M. pallidula* (Baly, 1874) [62]; and *M. wilcoxi* Gressitt & Kimoto, 1965 [63]. All newly sequenced mitochondrial genomes were submitted to GenBank, with accession numbers OR582724-OR582727 (Table 1). The four newly obtained sequences were circled and ranged from 15,866 bp (*M. pallidula*) to 16,965 bp (*M. wilcoxi*) in length, with significant variation in the size of the species mainly occurring in the control regions. All newly sequenced mitogenomes contained 37 genes (13 *PCGs*, 22 *tRNAs*, and 2 *rRNAs*) and a large non-coding region (control region), which is usually present in most insect mitochondrial genomes (Figure 1). Overall, the mitogenomic structure and nucleotide composition of these four species exhibited typical features of the family Chrysomelidae. Among these 37 genes, 9 genes from 13 *PCGs* and 14 genes from *tRNAs* were transcribed on the majority strand (J-strand), with the remaining genes oriented on the minority strand (N-strand). The four new mitogenomes only had a few overlaps between their detected genes, and the organization was very compact (Table S2). A UUU anticodon in *tRNA-Lys*, unique to Chrysomeloidea, was derived in the four newly sequenced mitogenomes of *Monolepta,* which is consistent with previous research [30,35,37].

The AT content of the newly sequenced mitogenomes exhibited a high degree of similarity in nucleotide composition (Table 2). All new mitogenomes had a significant bias of the total nucleotide composition toward A and T: 79.1% in *M. bicavipennis*; 79.3% in *M. cavipennis*; 79.8% in *M. wilcoxi*; and 78.8% in *M. pallidula* (Table 2). The skew metrics of four mitogenomes showed that AT-skew was positive and GC-skew was negative in *PCGs*, *rRNAs*, *tRNAs*, and control regions. The skew analysis indicated that the obvious bias was toward the use of A and C in the whole genomes (Table 2).

**Figure 2:**
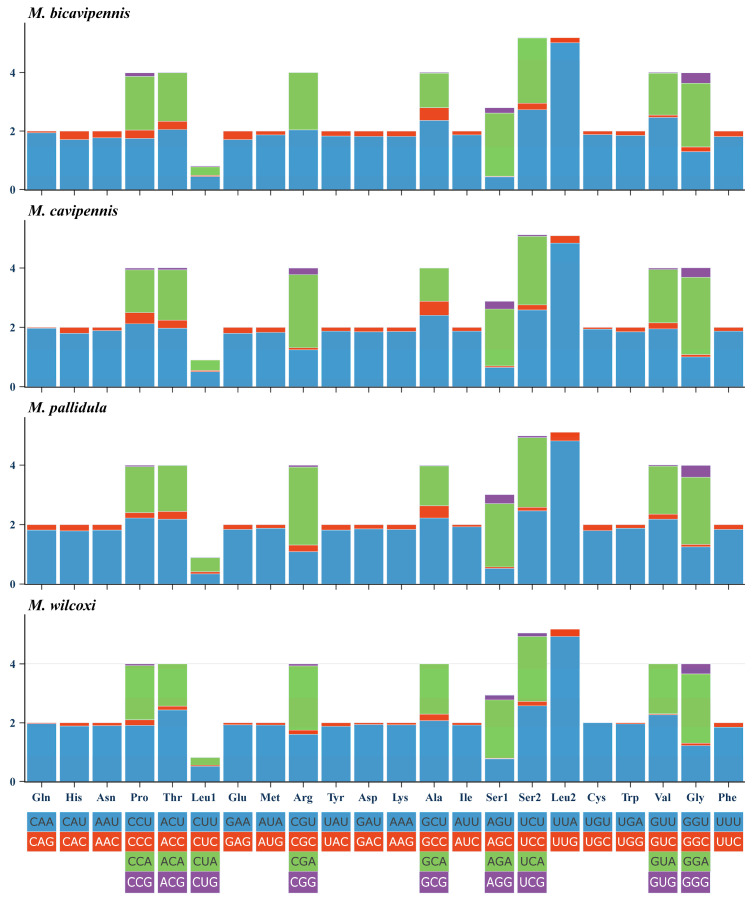
Relative synonymous codon usage (RSCU) of the four new mitogenomes.

The lengths of 13 *PCGs* were not significantly different, ranging from 11,118 bp to 11,121 bp. For the four new mitogenomes, the details for start and stop codons of protein-coding genes can be seen in Table S2. Except for the *ND1* gene starting with TTG, the rest of the *PCGs* started with ATN, all stop codons of 13 *PCGs* were TAA/TAG or just one single T. There is a very high similarity between the relative synonymous codon usage (RSCU) of the four sequenced mitogenomes and that of other previously determined beetles, and shows a codon usage bias (Figure 2): A and U were more frequently used than G and C. UUA, UCU, CGA, and GGA were the most frequently used codons.

**Figure 3:**
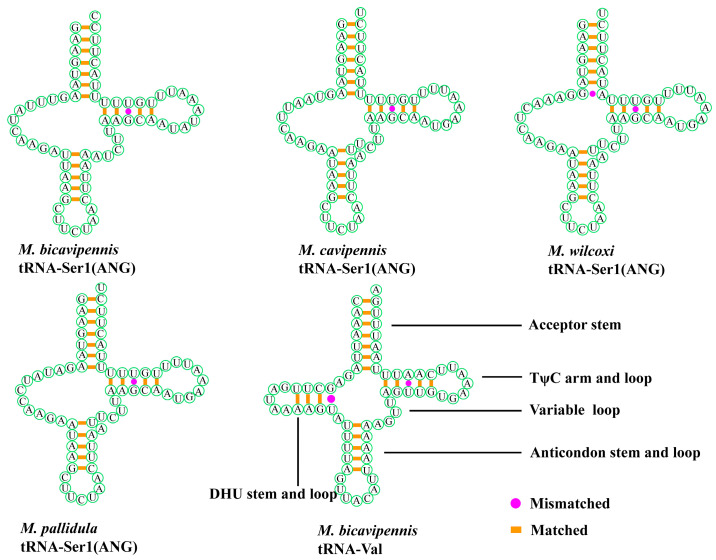
The secondary structure of *tRNA-Ser1* (AGN) in the four newly determined mitogenomes, and the predicted secondary structure of *tRNA-Val* in the *M. bicavipennis* mitogenome. The pink circle represents a mismatched base, and the orange square represents a matched base.

The location and characteristics of the two *rRNA* genes are similar to those of previously studied beetles. The 16S *rRNA* gene is located between *tRNA-Leu* (TAG) and *tRNA-Val*. The 12S *rRNA* gene is located between *tRNA-Val* and the control region. The arrangement of the *tRNA* genes of the four new sequenced species is very conserved. The secondary structure of all 22 *tRNAs* is folded into the typical cloverleaf structure, except *tRNA-Ser1* (AGN). Compared with the typical cloverleaf structure, *tRNA-Ser1* (AGN) lacks the DHU-stem, with several unmatched base pairs in the anticodon stem (Figure 3). More information on the four newly observed mitochondrial structures can be seen in the Supplementary Data (Table S2).

**Figure 4:**
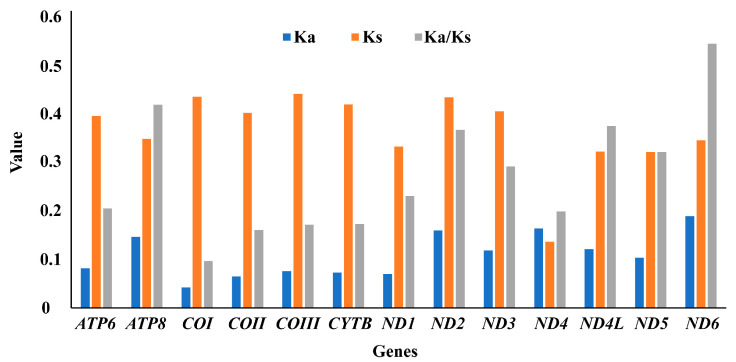
Non-synonymous (Ka) to synonymous (Ks) substitution rates of 13 *PCGs* among four sequenced species.

The average ratio of nonsynonymous (Ka) to synonymous (Ks) substitution could be used to estimate non-neutral changes relative to neutral changes and the degree of the selective pressure of a *PCG* [64]. In this study, the Ka/Ks substitution ratios of 13 *PCGs* were less than one, and ranged from 0.09672 (*COI*) to 0.54603 (*ND6*) (Figure 4). The results demonstrated that all *PCGs* were under purifying selection. The evolution rate of 13 *PCGs* was as follows: *ND6* > *ATP8* > *ND4L* > *ND2* > *ND5* > *ND3* > *ND1* > *ATP6* > *ND4* > *CYTB* > *COIII* > *COII* > *COI*. Among them, *COI* showed the lowest evolution rate, while *ND6* and *ATP8* exhibited a faster evolutionary rate and greater diversity than other *PCGs*.

**Figure 5:**
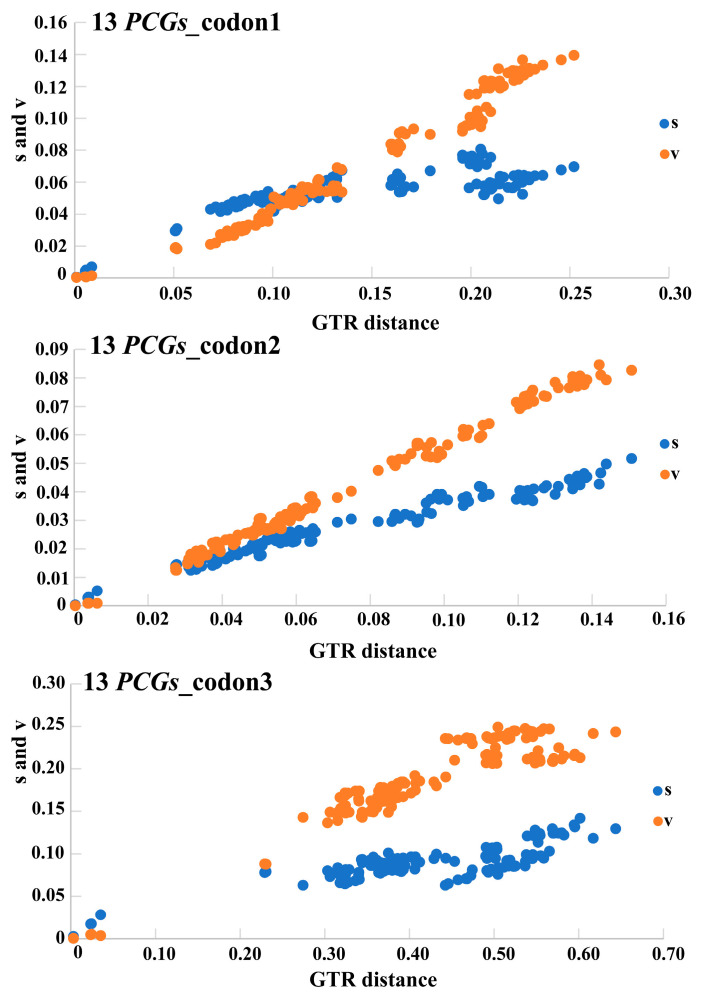
The chart of substitution saturation for the three different mitogenomes’ datasets. The plots show uncorrected pairwise divergences in transitions (s) (blue) to transversions (v) (orange) compared with divergences calculated by GTR model.

Substitution saturation testing reduces the amount of phylogenetic information contained in sequences and affects phylogenetic analyses involving deep branches. The substitution saturation of three datasets (13 *PCGs*_codon1, 13 *PCGs*_codon2, 13 *PCGs*_codon3) was assessed using DAMBE v.7. All the analyzed results showed a lower ISS value (simple index of substitution saturation) than ISS.c value (critical ISS value) (*p* < 0.05), which indicated all four datasets were not saturated (Figure 5). All those data types are feasible to use in phylogenetic analyses.

### 3.2. The Phylogeny of Monolepta

To verify the phylogenetic position of *M. bicavipennis*, *M. cavipennis*, *M. wilcoxi*, and *M. pallidula* within *Monolepta*, we used three datasets including 15 genes, 13 *PCGs*_AA, and *ND1* to reconstruct the phylogenetic relationships using IQ-TREE and Phylobayes methods.

**Figure 6:**
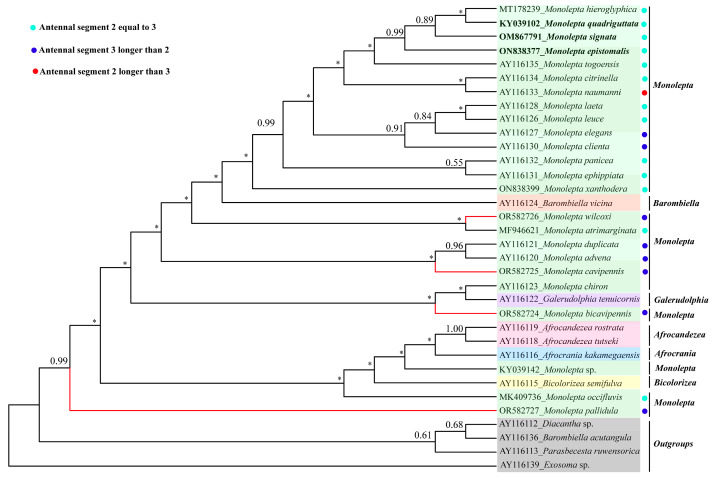
Phylogenetic tree reconstructed by Bayesian inference method based on *ND1* gene (34 species) under CAT-GTR model. Newly sequenced species in this study are highlighted in red color. The numbers above nodes are Bayesian posterior probabilities. Tips of synonymous species are highlighted in black color. Asterisk indicates that the bootstrap value of the node is lower than 0.50. Different colored backgrounds represent the different genera or outgroups.

The *ND1* dataset, including 34 taxa, was mainly pulled from the previous report by Stapel et al. [8], which was used to reconstruct the phylogenetic trees using the above two methods. The results showed that the topologies of IQ-TREE and Phylobayes tree were similar (Figure 6 and Figure S1). *Monolepta* was polyphyletic, with *Afrocandezea*, *Afrocrania*, *Barombiella*, and *Pseudocrania* emerging inside the Phylobayes tree. The newly sequenced Chinese species were divided into several distantly related branches. *M. wilcoxi* and *M. atrimarginata* were sister groups with low bootstrap value support; *M. cavipennis* and (*M. advena* + *M. duplicata*) and *M. bicavipennis* and (*Galerudolphia tenuicornis* + *M. chiron*) were sister groups, respectively; and *M. pallidula* was separated from all *Monolepta* species. Additionally, *M. quadriguttata*, *M. hieroglyphica*, and *M. signata* formed a clade with 89% bootstrap values, which is consistent with the results of Ge et al. [28].

**Figure 7:**
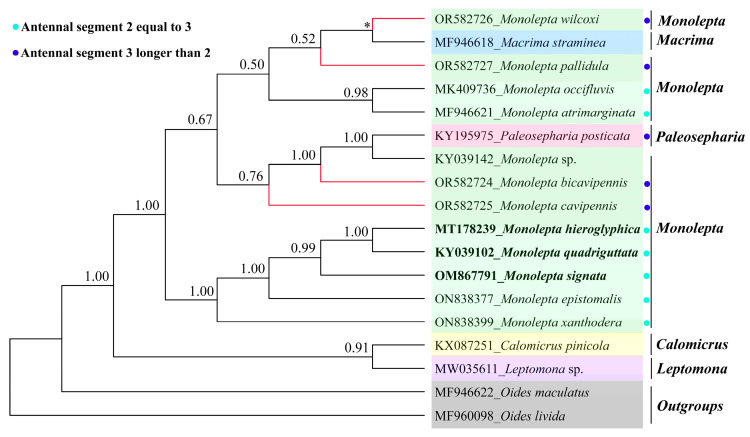
Phylogenetic tree reconstructed by Bayesian inference method based on 13 *PCGs*-AA (18 species) under CAT-GTR model. Newly sequenced species in this study are highlighted in red color. The numbers above nodes are Bayesian posterior probabilities. Tips of synonymous species are highlighted in black color. Asterisk indicates that the bootstrap value of the node is lower than 0.50. Different colored backgrounds represent the different genera.

For the 15 genes dataset (13 *PCGs* + 2 *rRNAs*) and 13 *PCGs*_AA, the 18 taxa were mainly pulled from NCBI and newly sequenced mitogenomes. The topologies of trees in Phylobayes and IQ-TREE were similar, except for the position of *Macrima straminea*, *M. atrimarginata*, and *M. occifluvis* (Figure 7 and Figures S2–S4). All *Monolepta* species were divided into two clades based on two datasets using different tree building methods (Figure 7 and Figures S1, S2 and S4), except for *M. occifluvis* and *M. atrimarginata*, which separated from all *Monolepta* species in the Phylobayes tree (Figure S3). The results showed that “Monoleptites” was a monophyletic group, while *Monolepta* was polyphyletic, splitting into several distant branches nested within *Paleosepharia* and *Macrima*. The four newly sequenced species had close relationships with *Paleosepharia. M. quadriguttata*, *M. hieroglyphica*, and *M. signata* formed one branch with a 100% bootstrap value and were proposed to be one species by Ge et al. [28] which was sister to *M. epistomalis* with the support of a 100% bootstrap value. The species of *Monolepta* with equal lengths were divided into two clades.

## 4. Discussion and Conclusions

In this study, the complete mitogenomes of four species of *Monolepta*, *M. bicavipennis*, *M. cavipennis*, *M. wilcoxi*, and *M. pallidula*, were sequenced successfully. All newly sequenced mitogenomes had similar structural characteristics and nucleotide compositions to previously published Chrysomelidae data. By combining the available mitogenomes of “Monoleptites” (18 taxa), their base compositions were calculated herein, and the results showed that all four mitogenomes were obviously biased towards A and T, which were similar to other beetles. The results of the ratio of nonsynonymous to synonymous substitution indicated that *ND6* had the highest evolution rate, while *COI* displayed the lowest evolution rate, which is different from other leaf beetles. Hebert et al. [65] argued that a *COI*-based DNA barcoding identification system could be developed for all animals, that is, *COI* divergences can serve as an effective tool in species recognition; in this recognition system, intraspecific divergences are rarely greater than 2% and most are less than 1%. In the previous study, the *COI* (766 bp extracted according to primers of LCO and HCO) intraspecific divergences of *M. hieroglyphica*, *M. quadriguttata*, and *M. signata* were shown to be 1.3%, while those of *ND1* were less than 1% (0.03%), and *M. hieroglyphica* and *M. quadriguttata* were confirmed to be synonyms of *M. signata* [28]. However, the divergences of the related, near-allied species of *M. signata* and *M. wilcoxi* was 12% for *COI*-barcode and 17% for *ND6*. Taking the highest interspecific evolution rate and lower intraspecific divergences of *ND6*, we propose that *ND6* may be enabling the discrimination of closely allied species in *Monolepta* as an effective DNA marker. 

Before constructing the phylogenetic tree, we tested the substitution saturation of 13 *PCGs*_codon1, 13 *PCGs*_codon2, and 13 *PCGs*_codon3. The results indicated all those data types were feasible to use to construct phylogenetic relationships. The phylogenetic relationship of the section “Monoleptites” was reconstructed based on three different datasets (15 genes, 13 *PCGs*_AA and *ND1*) using two different methods (IQ-TREE and Phylobayes). The topology of the phylogenetic tree based on *ND1* was very similar to the topology built by Stapel et al. [8], based on *ND1* and *ITS2*, which indicated that *Monolepta* was a non-monophyletic group. The Chinese distributed species of *Monolepta* were split into several clades and grouped with the African *Monolepta* species. *Barombiella*, *Afrocrania*, and *Afrocandezea* are restricted to African genera, which created one clear clade nested within *M. occifluvis* and *Monolepta* sp. with weak support. The phylogenetic inference based on the mitogenome showed a neater topology and higher node-supported value than that based on *ND1* data. The mitogenomic data showed greater power to resolve most expected main clades than the *ND1* gene at the suprageneric and species levels; this is mainly due to the larger number of variable characters, whereas each site also contains more information on average than *ND1*. The phylogenetic analyses based on mitogenomic data revealed that *Monolepta* was a non-monophyletic group. All the newly sequenced species whose antennal segment 2 was shorter than 3 had near relationships with *Paleosepharia*, and also had near relationships with *Macrima*. *Monolepta* was a very complicated group. Wilcox [66] was especially aware of the many inconsistent allocations of species to *Monolepta*. He commented that this genus needed to be revised and many species should be transferred to other genera. Lee [67] stated that some species of the genus *Monolepta* could be members of *Paleosepharia* and transferred four *Monolepta* species to *Paleosepharia*. In addition, the current study showed that the species whose antennal segments 2 and 3 are equal were divided into two clades. Wagner [9] re-described the type species of *Monolepta* and stated that the true “*Monolepta*” are those with a second and third antennomere of the same length. However, the current study supported that the characteristic of “antennal segment 2 as long as 3” of the true “*Monolepta*” evolved multiple times in several subgroups. So, to better understand the status of *Monolepta* and the suprageneric phylogenetic relationships at the species level, the revision of *Monolepta*’s relationship with related genera should be conducted first. Then, more taxon sampling and more molecular markers will be required in the future.

## Figures and Tables

**Table 1:** The information of 18 examined species with NCBI accession numbers and their references.

Subfamily	Tribe	Species	Length (bp)	Acc. No.	References
Galerucinae	Luperini	*Calomicrus pinicola*	15,436	KX087251	unpublished
Galerucinae	Luperini	*Leptomona* sp.	16,697	MW035611	unpublished
Galerucinae	Luperini	*Macrima straminea*	15,567	MF946618	[30]
Galerucinae	Luperini	*Monolepta atrimarginata*	15,143	MF946621	[30]
Galerucinae	Luperini	*Monolepta bicavipennis*	16,672	OR582724	this study
Galerucinae	Luperini	*Monolepta cavipennis*	16,965	OR582725	this study
Galerucinae	Luperini	*Monolepta epistomalis*	15,161	ON838377	unpublished
Galerucinae	Luperini	* Monolepta hieroglyphica	16,299	MT178239	[58]
Galerucinae	Luperini	*Monolepta occifluvis*	15,998	MK409736	unpublished
Galerucinae	Luperini	* Monolepta quadriguttata	16,130	KY039102	[59]
Galerucinae	Luperini	*Monolepta pallidula*	15,866	OR582727	this study
Galerucinae	Luperini	* Monolepta signata	16,329	OM867791	unpublished
Galerucinae	Luperini	*Monolepta* sp.	15,792	KY039142	[59]
Galerucinae	Luperini	*Monolepta wilcoxi*	16,012	OR582726	this study
Galerucinae	Luperini	*Monolepta xanthodera*	14,782	ON838399	unpublished
Galerucinae	Luperini	*Paleosepharia posticata*	15,729	KY195975	unpublished
Galerucinae	Oidini	*Oides livida*	16,127	MF960098	[30]
Galerucinae	Oidini	*Oides maculatus*	15,089	MF946622	[30]

Note: * The synonymous species of *M. quadriguttata, M. signata,* and *M. hieroglyphica* were not combined because it is better to keep the original names they had when they were submitted.

**Table 2:** Base composition of the four mitogenomes.

Species	Whole Mitogenome			Protein-Coding Genes	12S *rRNA* Genes	16S *rRNA* Genes	Control Region
	A + T%	A + T%	AT-Skew	GC-Skew	A + T%	A + T%	A + T%
*M. bicavipennis*	79.1	77.6	−0.145	0.003	81.2	83.0	83.4
*M. cavipennis*	79.3	77.6	−0.142	0.017	81.7	82.0	83.1
*M. pallidula*	78.8	77.3	−0.143	0.015	80.7	83.0	85.9
*M. wilcoxi*	79.8	78.4	−0.143	0.018	83.3	83.1	82.5

## Data Availability

The following information was supplied regarding the availability of DNA sequences: the new mitogenomes are deposited in GenBank of NCBI and the accession numbers are OR582724-OR582727.

## Supplementary Materials

The following supporting information can be downloaded at: https://www.mdpi.com/article/10.3390/insects15010050/s1. Figure S1. Phylogenetic tree reconstructed with IQ-TREE method based on *ND1* gene (34 species) under MFP + MERGE model. Newly sequenced species in this study are highlighted in red color. The numbers above nodes in the tree are the SH-aLRT (left) and bootstrap values (right). Tips of synonymous species are highlighted in black color. Different colored backgrounds represent the different genera or outgroups. Figure S2. Phylogenetic tree reconstructed with IQ-TREE method based on amino acids of 13 *PCGs*-AA (18 species) under MFP + MERGE model. Newly sequenced species in this study are highlighted in red color. The numbers above nodes in the tree are the SH-aLRT (left) and bootstrap values (right). Tips of synonymous species are highlighted in black color. Different colored backgrounds represent the different genera. Figure S3. Phylogenetic tree reconstructed using Bayesian inference method based on 15 genes (*13 PCGs* + 2 *rRNAs*) (18 species) under CAT-GTR model. Newly sequenced species in this study are highlighted in red color. The numbers above nodes are Bayesian posterior probabilities. Tips of synonymous species are highlighted in black color. Asterisk indicates that the bootstrap value of the node is lower than 0.50. Different colored backgrounds represent the different genera. Figure S4. Phylogenetic tree reconstructed with IQ-TREE method based on 15 genes (13 *PCGs* + 2 *rRNAs*) (18 species) under MFP + MERGE model. Newly sequenced species in this study are highlighted in red color. The numbers above nodes in the tree are the SH-aLRT (left) and bootstrap values (right). Tips of synonymous species are highlighted in black color. Different colored backgrounds represent the different genera. Table S1. Samples and register gene information (*ND1* data). Table S2. Organization of four newly sequenced mitogenomes of *Monolepta*.
