# Supplementary material for: Characterization of Four Complete Mitogenomes of Monolepta Species and Their Related Phylogenetic Implications
Source: Insects. 2024 Jan 11;15(1):50. doi: 10.3390/insects15010050 (PMC10816406; doi:10.3390/insects15010050)
Supplement: Supplementary file 1 [file insects-15-00050-s001.zip › Figure S2.pdf]

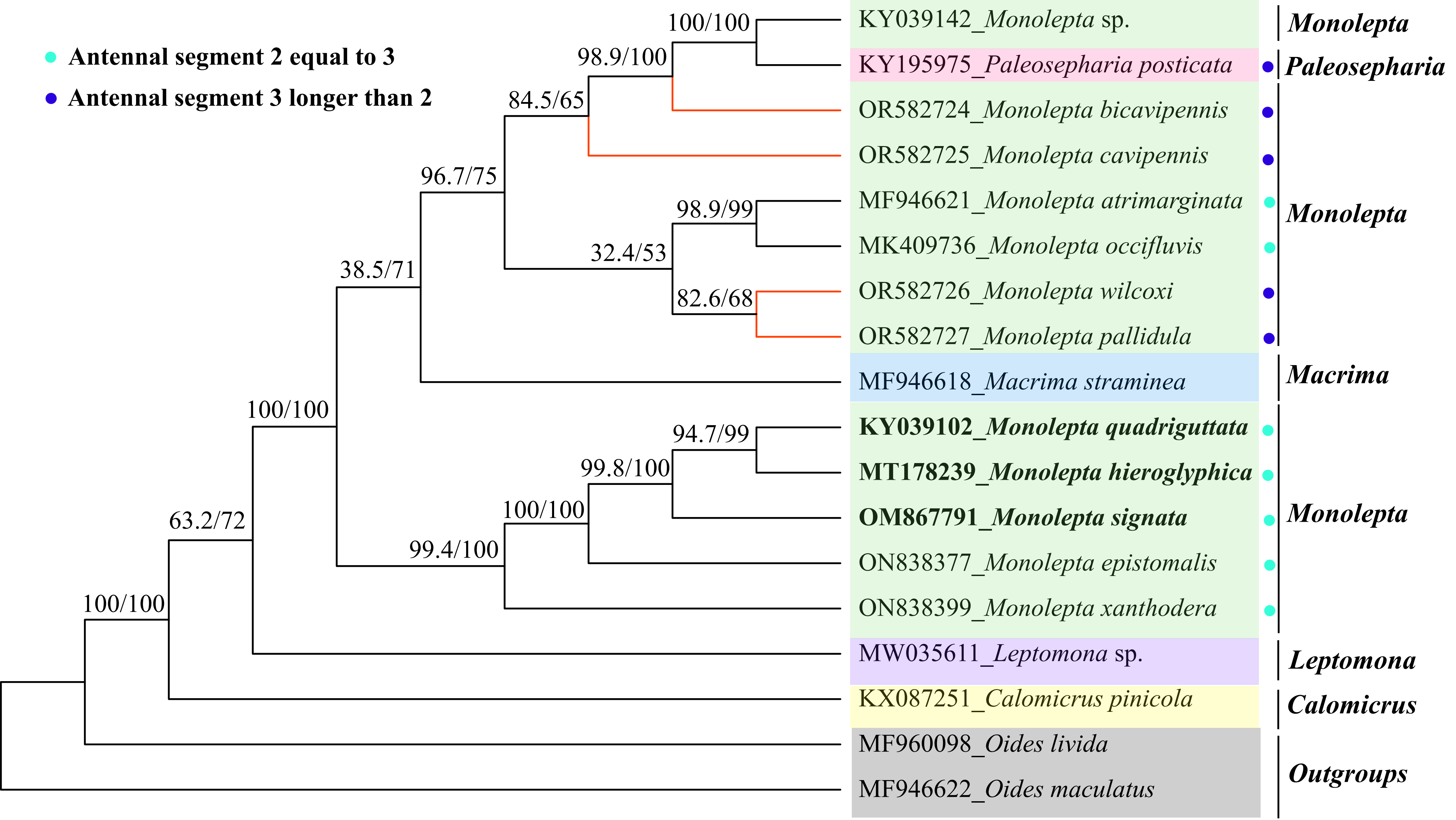

Figure S2. Phylogenetic tree reconstructed with IQ-TREE method based on amino acids of 13 *PCGs*-AA (18 species) under MFP + MERGE model. Newly sequenced species in this study are highlighted in red color. The numbers above nodes in the tree are the SH-aLRT (left) and bootstrap values (right). Tips of synonymous species are highlighted in black color. Different colored back-grounds represent the different genera.
