# Supplementary material for: Characterization of Four Complete Mitogenomes of Monolepta Species and Their Related Phylogenetic Implications
Source: Insects. 2024 Jan 11;15(1):50. doi: 10.3390/insects15010050 (PMC10816406; doi:10.3390/insects15010050)
Supplement: Supplementary file 1 [file insects-15-00050-s001.zip › Figure S3.pdf]

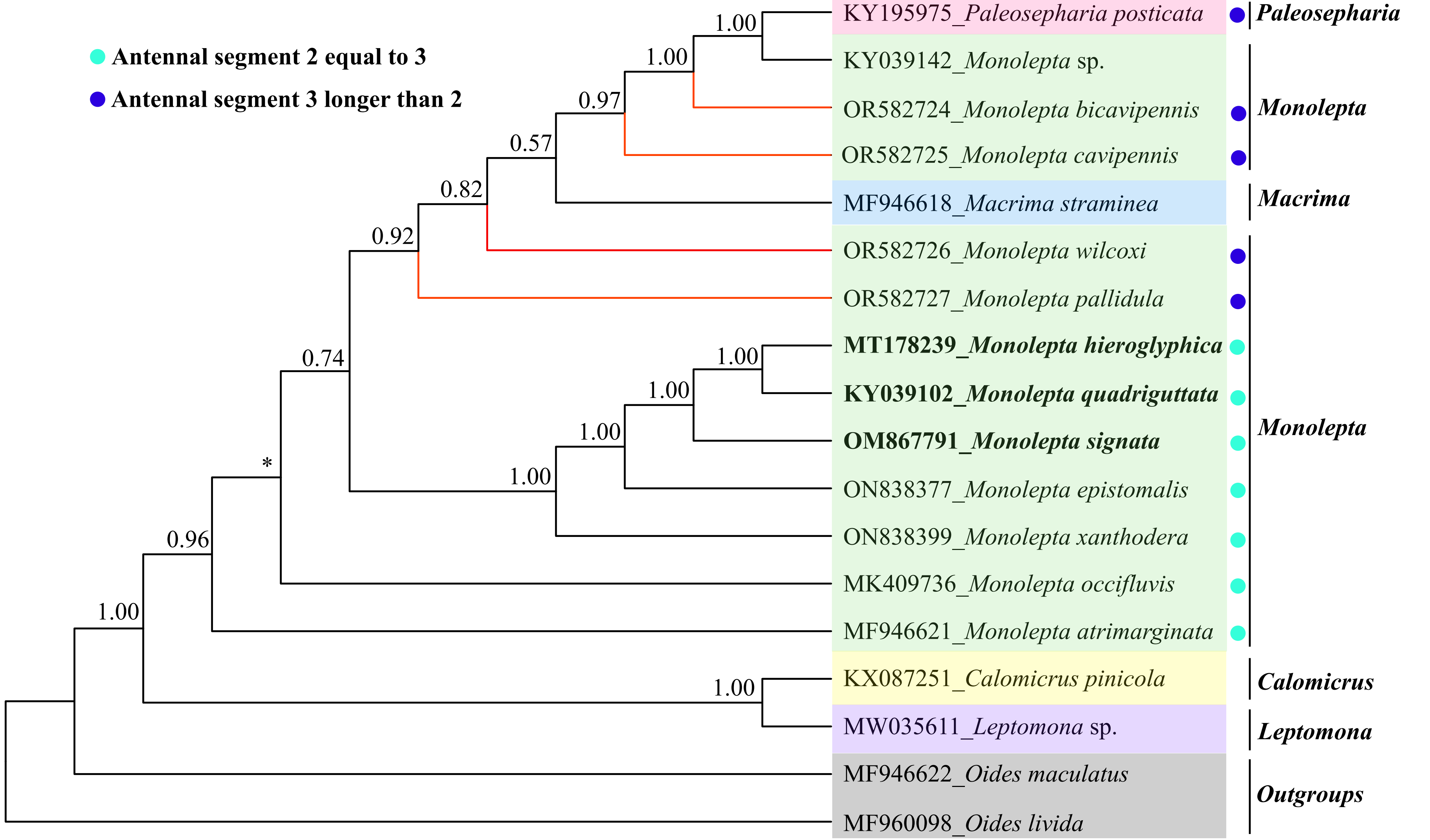

Figure S3. Phylogenetic tree reconstructed using Bayesian inference method based on 15 genes (13 *PCGs* + 2 *rRNAs*) (18 species) under CAT-GTR model. Newly sequenced species in this study are highlighted in red color. The numbers above nodes are Bayesian posterior probabilities. Tips of synonymous species are highlighted in black color. Asterisk indicates that the bootstrap value of the node is lower than 0.50. Different colored backgrounds represent the
