# Supplementary material for: Characterization of Four Complete Mitogenomes of Monolepta Species and Their Related Phylogenetic Implications
Source: Insects. 2024 Jan 11;15(1):50. doi: 10.3390/insects15010050 (PMC10816406; doi:10.3390/insects15010050)
Supplement: Supplementary file 1 [file insects-15-00050-s001.zip › Figure S4.pdf]

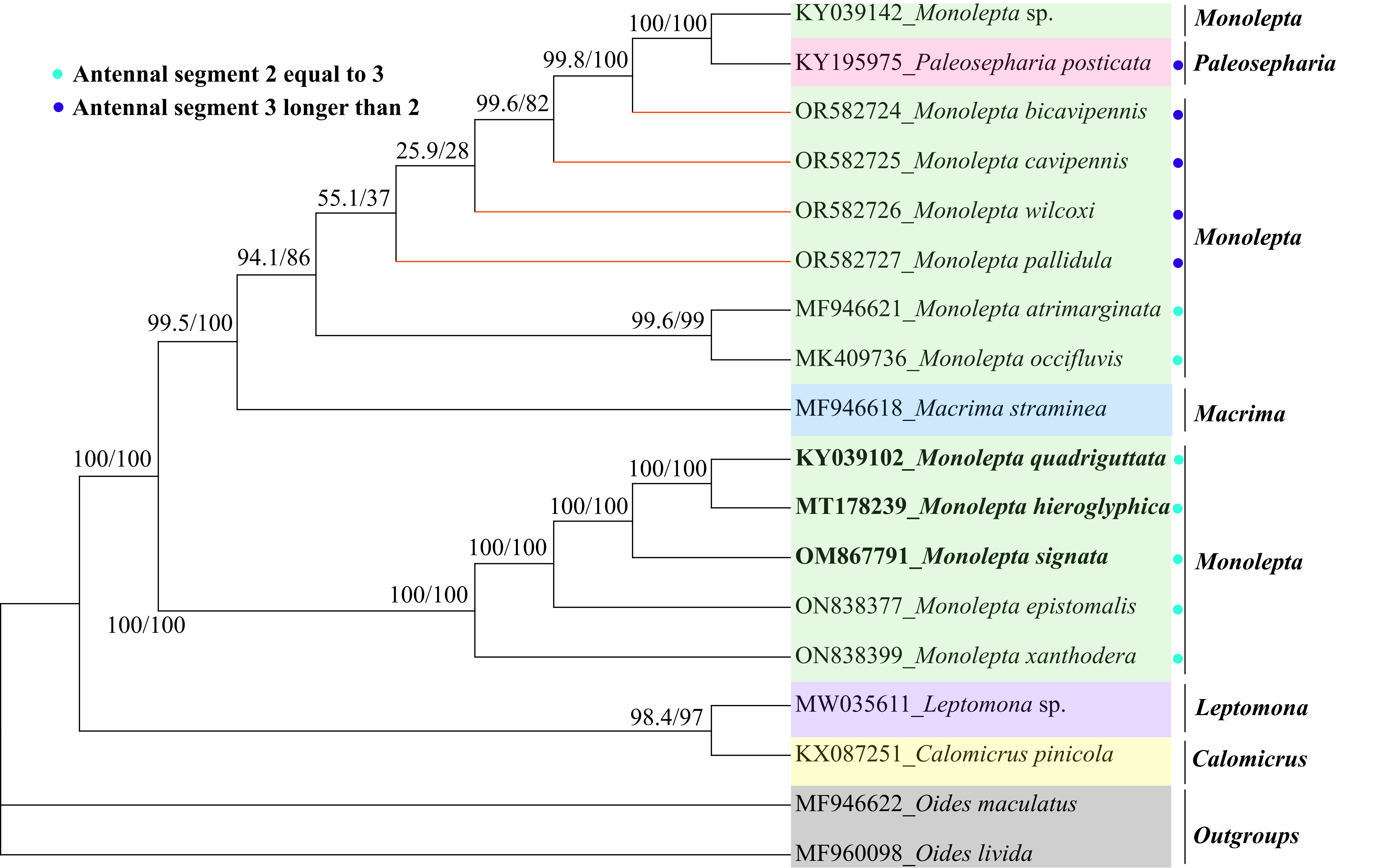

Figure S4. Phylogenetic tree reconstructed with IQ-TREE method based on 15 genes (13 *PCGs* + 2 *rRNAs*) (18 species) under MFP + MERGE model. Newly sequenced species in this study are highlighted in red color. The numbers above nodes in the tree are the SH-aLRT (left) and bootstrap values (right). Tips of synonymous species are highlighted in black color. Different colored backgrounds repre-sent the different genera.
