# Supplementary material for: Characterization of Four Complete Mitogenomes of Monolepta Species and Their Related Phylogenetic Implications
Source: Insects. 2024 Jan 11;15(1):50. doi: 10.3390/insects15010050 (PMC10816406; doi:10.3390/insects15010050)
Supplement: Supplementary file 1 [file insects-15-00050-s001.zip › Table S1.pdf]

Table S1. Samples and register gene information (*ND1* data).

Taxa	Acc. No.	Collection place	Ref.
Afrocandezea rostrata	AY116119	Kenya (Kaimosi)	Stapel *et al*. (2008)
Afrocandezea tutseki	AY116118	Kenya(Kakamega Forest)	Stapel *et al*. (2008)
Afrocrania kakamagaensis	AY116116	Kenya (Kakamega Forest)	Stapel *et al*. (2008)
Barombiella acutangula	AY116136	Kenya (Kakamega Forest)	Stapel *et al*. (2008)
Barombiella vicina	AY116124	Kenya (Kakamega Forest)	Stapel *et al*. (2008)
Diacantha sp.	AY116112	Kenya (Teita Hills)	Stapel *et al*. (2008)
Exosoma sp.	AY116139	Kenya (Teita Hills)	Stapel *et al*. (2008)
Galerudolphia tenuicornis	AY116122	Ivory Coast (Comoe NP)	Stapel *et al*. (2008)
Monolepa advena	AY116120	Kenya (Teita Hills)	Stapel *et al*. (2008)
Monolepta citrinella	AY116134	Kenya (Kakamega Forest)	Stapel *et al*. (2008)
Monolepta chiron	AY116123	Kenya (Kakamega Forest)	Stapel *et al*. (2008)
Monolepta clienta	AY116130	Ivory Coast (Comoe NP)	Stapel *et al*. (2008)
Monolepta deleta	AY116128	Kenya (Mt. Kenya)	Stapel *et al*. (2008)
Monoleptoides duplicata	AY116121	Kenya (Kakamega Forest)	Stapel *et al*. (2008)
Monolepta elegans	AY116127	Kenya (Aberdare Mountains)	Stapel *et al*. (2008)
Monolepta ephippiata	AY116131	Kenya (Mpala)	Stapel *et al*. (2008)
Monolepta leuce	AY116126	Kenya (Aberdare Mountains)	Stapel *et al*. (2008)
Monolepta naumanni	AY116133	Kenya (Kakamega Forest)	Stapel *et al*. (2008)
Monolepta panicea	AY116132	Ivory Coast (Comoe NP)	Stapel *et al*. (2008)
Monolepta togoensis	AY116135	Kenya (Kakamega Forest)	Stapel *et al*. (2008)
Parasbecesta ruwensorica	AY116113	Kenya (Kakamega Forest)	Stapel *et al*. (2008)
Pseudocrania semifulva	AY116115	Kenya (Kakamega Forest)	Stapel *et al*. (2008)
Monolepta atrimarginata	MF946621	China, Guizhou, Zunyicity	Nie *et al*. (2018)
Monolepta hieroglyphica	MT178239	unknow	Jiang *et al*. (2019)
Monolepta occifluvis	MK409736	unknow	unpublished
Monolepta quadriguttata	KY039102	unknow	Song *et al*. (2018)
Monolepta signata	OM867791	unknow	unpublished
Monolepta sp.	KY039142	unknow	Song *et al*. (2018)
Monolepta bicavipennis	OR582724	China, Shaanxi, Qinling	this study
Monolepta cavipennis	OR582725	China, Hainan, Jianfengling	this study
Monolepta wilcoxi	OR582726	China, Guizhou, Zunyicity	this study
Monolepta pallidula	OR582727	China, Anhui, Jinzhai	this study
Monolepta epistomalis	ON838377	unknow	unpublished
Monolepta xanthodera	ON838399	unknow	unpublished
