# Supplementary material for: Characterization of Four Complete Mitogenomes of Monolepta Species and Their Related Phylogenetic Implications
Source: Insects. 2024 Jan 11;15(1):50. doi: 10.3390/insects15010050 (PMC10816406; doi:10.3390/insects15010050)
Supplement: Supplementary file 1 [file insects-15-00050-s001.zip › Table S2.pdf]

Table S2. Organization of four newly sequenced mitogenomes of *Monolepta*.

1. Organization of *Monolepta bicavipennis.*

Gene	Start	End	Strand	Length(bp)	Intergenic nucleotides	Start/stop codons
trnI	1	77	J	77	-	
trnQ	78	140	N	63	0	
trnM	140	207	J	68	-1	
nad2	208	1221	J	1014	0	ATT/TAA
trnW	1222	1275	J	54	0	
trnC	1276	1337	N	62	0	
trnY	1338	1401	N	64	0	
cox1	1394	2941	J	1548	-8	ATT/TAA
trnL	2942	3001	J	60	0	
cox2	3002	3689	J	688	0	ATT/T-
trnK	3690	3759	J	70	0	
trnD	3760	3824	J	65	0	
atp8	3825	3980	J	156	0	ATT/TAA
atp6	3974	4648	J	675	-7	ATG/TAA
cox3	4648	5437	J	790	-1	ATA/T-
trnG	5439	5501	J	63	1	
nad3	5502	5855	J	354	0	ATT/TAG
trnA	5854	5918	J	65	-2	
trnR	5919	5984	J	66	0	
trnN	5980	6046	J	67	-5	
trnS	6047	6114	J	68	0	
trnE	6115	6175	J	61	0	
trnF	6176	6238	N	63	0	
nad5	6239	7940	N	1702	0	ATT/T-
trnH	7941	8003	N	63	0	
nad4	8004	9336	N	1333	0	ATG/T-
nad4L	9330	9611	N	282	-7	ATG/TAA
trnT	9615	9678	J	64	3	
trnP	9679	9745	N	67	0	
nad6	9746	10243	J	498	0	ATT/TAA
cytb	10243	11382	J	1140	-1	ATG/TAG
trnS	11383	11464	J	82	0	
nad1	11465	12415	N	951	0	TTG/TAG
trnL	12417	12482	N	66	1	
rrnL	12483	13755	N	1273	0	
trnV	13756	13824	N	69	0	
rrnS	13825	14629	N	805	0	
AT-rich region	14630	16672		2043	0	

2. Organization of *Monolepta cavipennis.*

Gene	Start	End	Strand	Length(bp)	Intergenic nucleotides	Start/stop codons
trnI	1	103	J	103	-	
trnQ	104	172	N	69	0	
trnM	170	242	J	73	-3	
nad2	243	1256	J	1014	0	ATT/TAA
trnW	1257	1311	J	55	0	
trnC	1312	1373	N	62	0	
trnY	1374	1438	N	65	0	
cox1	1431	2978	J	1548	-8	ATT/TAA
trnL	2979	3038	J	60	0	
cox2	3040	3727	J	688	1	ATT/T-
trnK	3728	3797	J	70	0	
trnD	3798	3859	J	62	0	
atp8	3860	4015	J	156	0	ATT/TAA
atp6	4009	4683	J	675	-7	ATG/TAA
cox3	4683	5469	J	787	-1	ATG/T-
trnG	5471	5533	J	63	1	
nad3	5534	5887	J	354	0	ATC/TAG
trnA	5886	5951	J	66	-2	
trnR	5952	6016	J	65	0	
trnN	6012	6078	J	67	-5	
trnS	6079	6146	J	68	0	
trnE	6147	6211	J	65	0	
trnF	6212	6275	N	64	0	
nad5	6276	7977	N	1702	0	ATT/T-
trnH	7978	8040	N	63	0	
nad4	8041	9373	N	1333	0	ATG/T-
nad4L	9367	9648	N	282	-7	ATG/TAA
trnT	9652	9717	J	66	3	
trnP	9718	9783	N	66	0	
nad6	9784	10281	J	498	0	ATT/TAA
cytb	10281	11420	J	1140	-1	ATG/TAG
trnS	11421	11502	J	82	0	
nad1	11503	12453	N	951	0	TTG/TAG
trnL	12455	12520	N	66	1	
rrnL	12521	13800	N	1280	0	
trnV	13801	13871	N	71	0	
rrnS	13872	14680	N	809	0	
AT-rich region	14681	16965		2285	0	

3. Organization of *Monolepta wilcoxi.*

Gene	Start	End	Strand	Length(bp)	Intergenic nucleotides	Start/stop codons
trnI	1	103	J	103	-	
trnQ	117	185	N	69	13	
trnM	183	254	J	72	-3	
nad2	255	1268	J	1014	0	ATT/TAA
trnW	1269	1324	J	56	0	
trnC	1325	1386	N	62	0	
trnY	1387	1450	N	64	0	
cox1	1443	2990	J	1548	-8	ATT/TAA
trnL	2991	3051	J	61	0	
cox2	3052	3739	J	688	0	ATT/T-
trnK	3740	3809	J	70	0	
trnD	3810	3872	J	63	0	
atp8	3873	4028	J	156	0	ATT/TAA
atp6	4022	4696	J	675	-7	ATG/TAA
cox3	4696	5482	J	787	-1	ATG/T-
trnG	5484	5545	J	62	1	
nad3	5546	5899	J	354	0	ATT/TAG
trnA	5898	5963	J	66	-2	
trnR	5964	6029	J	66	0	
trnN	6025	6091	J	67	-5	
trnS	6092	6159	J	68	0	
trnE	6160	6220	J	61	0	
trnF	6221	6282	N	62	0	
nad5	6285	7986	N	1702	2	ATT/T-
trnH	7987	8049	N	63	0	
nad4	8050	9382	N	1333	0	ATG/T-
nad4L	9376	9657	N	282	-7	ATG/TAA
trnT	9661	9723	J	63	3	
trnP	9724	9788	N	65	0	
nad6	9789	10289	J	501	0	ATT/TAA
cytb	10289	11428	J	1141	-1	ATG/TAA
trnS	11430	11511	J	82	1	
nad1	11512	12462	N	951	0	TTG/TAG
trnL	12464	12528	N	65	1	
rrnL	12529	13807	N	1279	0	
trnV	13808	13878	N	71	0	
rrnS	13879	14688	N	810	0	
AT-rich region	14689	16012		1324	0	

4. Organization of *Monolepta pallidula.*

Gene	Start	End	Strand	Length(bp)	Intergenic nucleotides	Start/stop codons
trnI	1	70	J	70	-	
trnQ	71	139	N	69	0	
trnM	137	207	J	71	-3	
nad2	208	1221	J	1014	0	ATT/TAA
trnW	1222	1275	J	54	0	
trnC	1276	1338	N	63	0	
trnY	1339	1399	N	61	0	
cox1	1398	2945	J	1548	-2	ATT/TAA
trnL	2946	3005	J	60	0	
cox2	3006	3693	J	688	0	ATT/T-
trnK	3694	3763	J	70	0	
trnD	3764	3831	J	68	0	
atp8	3832	3987	J	156	0	ATT/TAA
atp6	3981	4655	J	675	-7	ATG/TAA
cox3	4655	5441	J	787	-1	ATG/T-
trnG	5443	5505	J	63	1	
nad3	5506	5859	J	354	0	ATT/TAA
trnA	5858	5924	J	67	-2	
trnR	5925	5989	J	65	0	
trnN	5985	6051	J	67	-5	
trnS	6052	6119	J	68	0	
trnE	6120	6180	J	61	0	
trnF	6181	6244	N	64	0	
nad5	6245	7946	N	1702	0	ATT/T-
trnH	7947	8012	N	66	0	
nad4	8014	9346	N	1333	1	ATG/T-
nad4L	9340	9621	N	282	-7	ATG/TAA
trnT	9625	9689	J	65	3	
trnP	9690	9754	N	65	0	
nad6	9755	10255	J	501	0	ATT/TAA
cytb	10255	11394	J	1140	-1	ATG/TAG
trnS	11395	11476	J	82	0	
nad1	11477	12427	N	951	0	TTG/TAG
trnL	12429	12494	N	66	1	
rrnL	12495	13773	N	1279	0	
trnV	13774	13844	N	71	0	
rrnS	13845	14648	N	804	0	
AT-rich region	14649	15866		1218	0	
